# The action effect was not affected by cognitive load

**DOI:** 10.3389/fnins.2025.1616974

**Published:** 2025-09-22

**Authors:** Guang Zhao, Yuhao Duan, Hanxu Wang, Rongtao Wu, Jichao Zhang

**Affiliations:** Faculty of Psychology, Tianjin Normal University, Tianjin, China

**Keywords:** action effect, cognitive load, N2pc, LPC, decoding

## Abstract

**Introduction:**

Actions are traditionally thought to be guided by cognition. A reverse pathway where action influences perception has been revealed through the “action effect”. The action effect refers to the acceleration of target search when targets share features with previously acted-upon stimuli.

**Methods:**

In this study, to further understand its cognitive mechanism, we investigated whether the action effect is modulated by cognitive load. Participants were instructed to press a key if the prime stimulus (a colored color word) matched a specified color. Otherwise, they were instructed to passively view the prime. The color of the prime was either congruent (no cognitive load) or incongruent (cognitive load) with its semantic meaning. The magnitude of the action effect between the two conditions was compared. Using EEG technology, we addressed the research gap concerning the neural mechanisms underlying the action effect.

**Results:**

Behaviorally, response times were shorter in the action condition compared to the no-action condition, confirming the presence of the action effect. Notably, the magnitude of the action effect was equivalent between the congruence and incongruence conditions. Electrophysiological data revealed that attentional priority for acted-on stimuli was enhanced, while the response selection process was delayed. Importantly, all neural markers—including N2pc, P300b, and late LPC—exhibited minimal differences between the congruent and incongruent conditions.

**Discussion:**

The findings provide robust evidence that the action effect remains intact in the presence of cognitive load. This not only advances our understanding of its underlying mechanisms but also provides theoretical guidance for its potential application conditions.

## 1 Introduction

The successful execution of daily activities fundamentally depends on the intricate coordination between perceptual processing and motor action. While the modulatory role of visual attention in guiding actions has been extensively documented (Woodworth, 1899), emerging evidence reveals a bidirectional relationship in which actions can reciprocally shape our perception of external stimuli. Research has shown that action readiness selectively enhances perceptual sensitivity to action-relevant stimulus features. For example, as individuals prepare to grasp an object, their sensitivity to features critical for grasping, such as the object’s orientation or size, is enhanced relative to action-irrelevant attributes such as color or luminance (Bekkering and Neggers, 2002; Wykowska et al., 2009).

Notably, studies reveal that even arbitrary actions, without inherent functional relevance to the task, can systematically bias perceptual processing. In the seminal study by Buttaccio and Hahn (2011), a two-task paradigm was employed. In each trial, a color word (the cue) was first presented, followed by a colored shape (the prime). The task required judging color congruency between the cue and prime, with participants instructed to press the spacebar for congruent trials and maintain passive fixation for incongruent trials. Following this decision phase, a search array appeared containing targets whose color matched the prime at chance levels, ensuring that the prime provided no predictive information about target features. Critical findings revealed significantly faster response times in valid trials (the target color matching the prime) compared to invalid trials, demonstrating a robust validity effect. Crucially, this perceptual facilitation emerged specifically following action execution, as no comparable effect was observed when participants passively viewed the prime without motor responses. This suggests that action execution selectively prioritizes the perceptual features of acted-upon stimuli, enhancing subsequent target detection for matching features (in this case, color). To date, the phenomenon has been observed in numerous studies and has been termed the action effect (Han et al., 2020; Robinson et al., 2018; Suh and Abrams, 2018; Wang et al., 2017; Wang et al., 2021; Weidler and Abrams, 2014, 2016, 2018; Weidler et al., 2018).

The action effect provides a distinctive example of how motor execution can retroactively influence cognitive processing, even when actions lack inherent functional relevance to the cognitive task. Understanding the mechanisms underlying this phenomenon—particularly how arbitrary motor responses can selectively enhance sensitivity to stimulus features—helps reveal the ways in which action-cognition-perception interactions occur. A recent study examined whether eye movement patterns during visual search are altered by preceding actions (Weidler et al., 2018). They found that following an action, the first saccade is more likely to go to the stimulus sharing chromatic features with action-relevant primes. In addition, fewer eye movements were required when targets shared features with acted-upon primes, suggesting enhanced attentional guidance toward action-congruent stimulus features. Furthermore, prolonged target fixation durations on valid compared to invalid trials implied potential action-induced delays in response selection processes following target identification. Notably, the persistence of action effects under conditions of pop-out search (where attentional guidance is presumed optimal) challenges conventional attentional explanations (Weidler and Abrams, 2016). Through a series of three rigorously controlled experiments, researchers consistently observed action-contingent performance enhancements, indicating potential modulation of early perceptual processing stages rather than later attentional mechanisms.

A prominent theoretical framework explaining the action effect is derived from biased competition models, according to which multiple stimuli compete for neural representation (Desimone and Duncan, 1995; Shapiro and Miller, 2011). Huffman and Pratt (2017) posited that action execution increases the perceptual weighting of task-relevant features, creating competitive advantages for prime-congruent stimuli during early visual processing. This resource-dependent account predicts graded attenuation of action effects under conditions of diminished cognitive capacity. However, alternative evidence suggests that these effects might reflect automatic processes. Drawing from established cognitive control and automaticity frameworks (Moors and De Houwer, 2006; Schneider and Shiffrin, 1977), true automaticity should exhibit both task-irrelevance immunity and resource independence. The action effect has been observed even when primes remain behaviorally irrelevant across both action and search tasks (Weidler and Abrams, 2014). Moreover, recent research incorporating continuous flash suppression techniques has demonstrated that the action effect persists even when primes are presented below the perceptual threshold, providing further evidence that the action effect is not driven by deliberate control (Suh and Abrams, 2018). Collectively, these findings suggest that action-induced feature prioritization is immune to task irrelevance, supporting the automaticity of the action effect. However, another critical aspect of automaticity remains unexamined. Specifically, it remains unclear whether the emergence of the action effect is constrained by cognitive resource availability. Since feature prioritization occurs during action execution, this question can be more precisely framed as follows: Does the magnitude of the action effect depend on the cognitive resources available during action execution?

To address this gap, the current study was conducted to investigate the influence of cognitive load on the action effect. Cognitive load was defined as the mental effort required for an individual to complete a task (Chandler and Sweller, 1991; Sweller, 1988). Under the condition of higher cognitive load, fewer cognitive resources are available for concurrent processing. In the present study, cognitive load was introduced during action execution through a Stroop-like manipulation (Jongen and Jonkman, 2011; Muke et al., 2021). In the classic color-word Stroop paradigm, participants experience response interference when the word meaning conflicts with the ink color (e.g., “red” printed in blue) compared to congruent trials (e.g., “red” printed in red) (MacLeod, 1991). Building on this well-established interference effect, our paradigm employed colored words as primes while omitting the pre-prime cue typically used in traditional action effect paradigms. Crucially, we manipulated prime congruency across two conditions: congruent primes matched both the word meaning and ink color (e.g., “red” in red), whereas incongruent primes exhibited a mismatch (e.g., “red” in blue). During the visual search task, the target color matched the prime color in the valid trials but not in the invalid trials. For the hypothesis, if the action effect does not require the involvement of cognitive resources, we expect the magnitude of the action effect to remain unchanged in the incongruence condition compared to the congruence condition. Otherwise, if the action effect is sensitive to cognitive load, we expect the magnitude of the action effect to be reduced, even diminished, in the incongruence condition.

Previous research has not explored the neural markers of action effects, which also limits our understanding of the underlying cognitive mechanisms. To address this gap, we simultaneously recorded electrophysiological (EEG) data while participants performed the task. We used the event-related potential (ERP) method to investigate which stage of the search is affected by the arbitrary action to promote performance. We primarily focused on two cognitive processes related to search: the attentional allocation process and the response execution process. The attentional allocation process was reflected by the N2 posterior contralateral (N2pc) component and P300b. The N2pc is a lateralized component reflecting attentional selection of peripheral stimuli (Luck and Hillyard, 1994a, 1994b). It is characterized by a more negative deflection in the contralateral hemisphere relative to lateralized stimuli compared to the ipsilateral hemisphere, occurring approximately 200-300 ms after search array onset. The amplitude of the N2pc component typically reaches its maximum at parietal electrodes. The P300b is a subcomponent of the late positive complex (LPC), which manifests as a positive component that occurs approximately 300 ms after stimulus onset with maximum amplitude at parietal electrodes, reflecting stimulus evaluation following early attentional selection (Nasman and Rosenfeld, 1990; Polich, 2000). Its amplitude increases as more attentional resources are allocated (Polich, 2007). The response selection process was reflected by the late part of the LPC, which typically appears approximately 500 ms after stimulus onset with maximum amplitude at parietal electrodes (Sutton and Ruchkin, 1984). The late LPC is believed to be related to response execution following target detection (Falkenstein et al., 1994). We also employed multivariate pattern analysis (MVPA) to: (1) identify neural representations of target validity in the search task (i.e., whether the target matched the color of the previously presented prime) and (2) determine how the neural representation of the target is influenced by validity. MVPA considers the relationships between multiple variables (in our case, signals from multiple electrodes) rather than treating them as independent measures. Therefore, this approach can detect distributed spatial patterns and demonstrates higher sensitivity compared to traditional univariate methods (Grootswagers et al., 2017). MVPA has been widely applied in EEG research (Duncan et al., 2023; Li et al., 2023).

## 2 Methods

### 2.1 Participants

A total of 28 participants (20 female individuals, mean age ± SD = 20.07 ± 1.83 years, range = 18 to 25 years, all right-handed) from Tianjin Normal University in China participated in our experiment. The sample size was chosen based on previous studies of the action effect, which typically included 24 participants (Wang et al., 2017; Weidler and Abrams, 2014; Hommel, 1998; Hommel, 2004). Of the original sample, two participants were excluded due to excessive artifacts, leading to more than 40% of segments being removed. Data from 26 individuals were included in the formal analysis. The participants received approximately 30 RMB as compensation for their involvement in the experiment. All participants had normal or corrected-to-normal visual acuity and provided written informed consent before the experiment. The study was approved by the local institutional ethics committee.

### 2.2 Apparatus

The experiment was carried out in a soundproof room. The participants were seated approximately 90 cm from the monitor. The control of the experimental procedure was achieved through the E-prime 3.0 software. The stimuli were presented on a 19-inch LCD monitor (1,024 × 768 pixels, 75 Hz). The participants were instructed to keep their heads still during the task to minimize artifacts and were encouraged to relax during breaks.

### 2.3 Stimulus

All stimuli were presented against a black (RGB: 0, 0, 0) background. The prime words (“red,” “yellow,” and “blue,” approximately 1.7° × 1.66°) appeared at the center of the screen. Note that the prime words were presented in Chinese in our experiment. The color of the prime words could be red (RGB: 255, 0, 0), yellow (RGB: 255,255,0), or blue (RGB: 176, 240, 0). The search items were letters (either “T” or “L,” approximately 1.9° × 1.9°) embedded within a colored circle (red, blue, or yellow, approximately 2.78° in diameter). In each search array, two items were located at two diagonal corners of an invisible square, with equal distance (approximately 5.2°) from the center of the screen. Among the two search items, one was the target with a letter “T” inside, which was randomly rotated 90° to the left or right. The other was the distractor with a letter “L” inside, which was randomly rotated by 0°, 90°, 180°, or 270°.

### 2.4 Design and procedure

**Figure 1:**
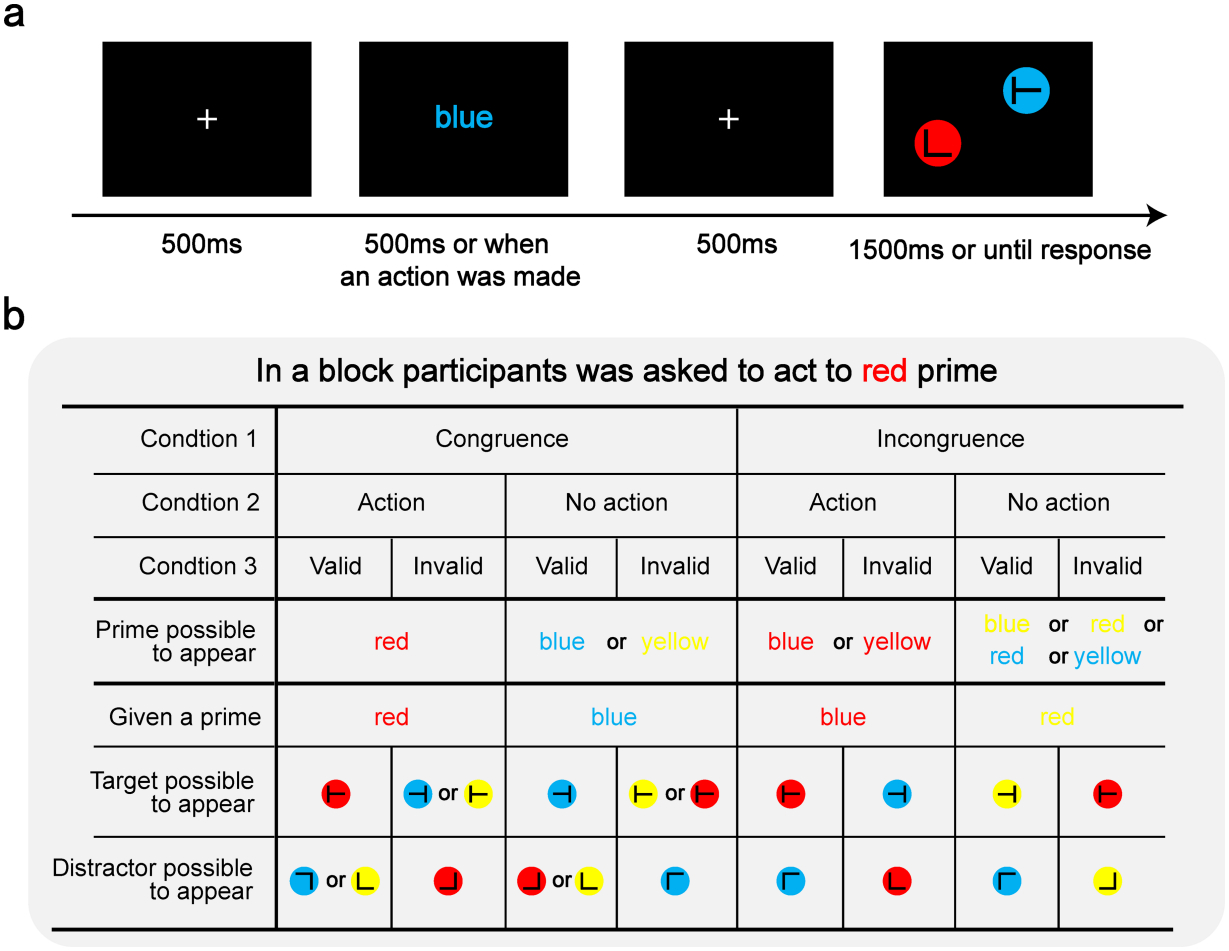
Design. **(a)** The procedure of a single trial. **(b)** Example of a block condition in which the participants responded to the red prime. Condition 1 indicates whether the color of the prime matched its semantic meaning. Condition 2 indicates whether the prime color matched the color the participants were asked to respond to. Condition 3 shows whether the search-for target color matched the previous prime color.

The experimental stimuli and procedure are shown in Figure 1a. Each trial began with a fixation cross presented for 500 ms. Then, the prime word was presented for 500 ms. The participants were instructed to press the “space” key with their left hand only when the prime words were printed in a given color. Otherwise, they were instructed to passively view the prime. The action-critical color varied across blocks, with explicit instructions indicating the target color displayed before each block. The prime disappeared after a 500 ms interval or once a response was detected, after which the search array was presented. The task was to find the target “T” and respond according to its degree of rotation. If the target T was tilted to the right, they had to press the right arrow button on a computer keyboard; if the target was tilted to the left, they had to press the left arrow button. The participants were instructed to respond using the right hand. They were encouraged to respond as quickly and accurately as possible in both the action and search tasks.

Crucially, the color and the word meaning of the prime were either congruent (e.g., the word “red” in red ink) or incongruent (e.g., the word “red” in blue ink). Each block contained eight congruent and eight incongruent trials, with equal distribution of action-required (four trials) and no-action (four trials) conditions within each congruence category. The target’s color either matched the prime’s color (valid trials) or differed (invalid trials), maintaining a 1:1 validity ratio across the conditions. Notably, the color–target relationships differed between the congruence conditions. In the congruent trials with the valid condition, the target matched the prime color and the distractors used the remaining colors. In the congruent trials with the invalid condition, the target used the non-prime colors and the distractor matched the prime color. However, in the incongruent trials with the valid condition, the target matched the prime color and the distractor matched the prime word meaning. In the incongruent trials with the invalid condition, the target matched the prime word meaning and the distractor matched the prime color. This design ensured the equivalent predictive value of the prime features for the target and distractor colors, eliminating potential search task advantages from prime processing.

After completing the practice trials, the participants proceeded to the formal experiment, which consisted of 30 blocks of 16 trials each. Action-critical colors were counterbalanced across three 10-block sequences (red, blue, yellow), with randomized block order. Figure 1b illustrates an example of the condition settings in a block where the participants were instructed to respond to the red prime.

### 2.5 EEG recording and preprocessing

EEG data were acquired using a 64-channel Neuroscan EEG acquisition device at a sampling rate of 1,000 Hz, with electrodes placed according to the international 10–20 system. Electrode impedance was maintained below 10kΩ throughout the recording session.

Offline preprocessing was performed using EEGLAB. The EEG data were down-sampled to 500 Hz, re-referenced to the average of the left and right mastoids, and filtered with a high-pass filter at 0.1 Hz and a low-pass filter at 30 Hz. The continuous EEG signal was then segmented into epochs spanning from 200 ms before search onset to 800 ms after search display onset. The segments containing significant artifacts were manually inspected and removed. Dysfunctional electrodes were manually identified. Independent component analysis (ICA) was then performed to correct for artifacts related to eye movements. The number of independent components was set to the number of functional electrodes minus one. For the identified independent components, we manually inspected all components and extracted those exhibiting topographic distributions characteristic of blink- or saccade-related signals. After removing these components, we re-examined the corrected data to confirm that ocular artifacts had been eliminated. The previously identified malfunctioning electrodes were then interpolated. Finally, epochs containing outliers were excluded. Specifically, epochs with voltages exceeding ±80 μV on any electrode were removed from subsequent analyses. Participants who had more than 40% of their segments removed were excluded from the analysis, resulting in the exclusion of two participants. The average number of trials retained among the participants included in the formal analysis was 476.65 ± 2.69 (min = 468). The average number of trials retained per condition among the participants included in the formal analysis was as follows: congruence condition: action-valid (59.38 ± 1.17, min = 56), action-invalid (59.5 ± 0.86, min = 57), no action-valid (59.23 ± 1.78, min = 57), and no action-invalid (59 ± 1.62, min = 54); incongruent conditions: action-valid (59.38 ± 0.98, min = 56), action-invalid (59.38 ± 0.98, min = 57), no action-valid (59.04 ± 1.37, min = 55), and no action-invalid (59.38 ± 1.02, min = 56).

### 2.6 Statistical analysis

#### 2.6.1 Behavioral data

For reaction time (RT) analyses, only trials with correct responses and RTs within ±2.5 standard deviations of each participant’s mean RT were included.

To investigate whether the action effect exists in both congruence and incongruence conditions, and whether the magnitude of the action effect differs between these conditions, three-way repeated-measures ANOVA was conducted on mean RTs, with congruency (congruent vs. incongruent), action type (action vs. no action), and validity (valid vs. invalid) as within-subject factors. The following analyses were performed when the three-way interaction was significant. First, simple interaction effect tests were conducted to examine whether action effects emerged under the congruence and incongruence conditions separately. Specifically, two-way repeated-measures ANOVA was conducted on mean RTs, with action type (action vs. no action) and validity (valid vs. invalid) as within-subject factors, separately for the congruence and incongruence conditions. Second, to further determine whether cognitive load affected the magnitude of the action effect, we calculated validity effects and conducted two-way repeated-measures ANOVA, with congruency (congruent vs. incongruent) and action type (action vs. no action) as within-subject factors, using the magnitude of the validity effect as the dependent variable. The validity effect was defined as the mean RTs for the invalid trials minus the mean RTs for the valid trials, computed separately for each combination of action type and congruency. Significant two-way interactions were followed up with simple effects analyses using the Bonferroni correction. Furthermore, to rule out potential confounding effects of speed-accuracy trade-offs on the interpretation of our results, identical analyses were conducted on accuracy data.

#### 2.6.2 Event-related potentials

For event-related potential (ERP) analyses, electrophysiological data were segmented into epochs from −200 to 800 ms relative to search display onset. The parieto-occipital electrodes PO7 and PO8 were selected for analyzing the N2pc component. Based on the visual inspection of the N2pc data, we selected a time window of 180–300 ms relative to stimulus onset (Luck and Hillyard, 1994a, 1994b). For the P300b and late LPC components, parieto-occipital electrodes (P3, P4, P7, P8, PO3, PO4, PO5, PO6, PO7, and PO8) were selected, with time windows of 300–500 ms and 500–700 ms, respectively (Falkenstein et al., 1994; Sutton and Ruchkin, 1984).

Mean amplitudes during the corresponding time windows were computed for each ERP component. Three-way repeated-measures ANOVA was conducted, with congruency (congruent vs. incongruent), action type (action vs. no action), and validity (valid vs. invalid) as within-subject factors, using the mean amplitude of each ERP component as the dependent variable. Similar follow-up analyses were conducted, as described in the Behavioral Data section. The validity effect for the N2pc component was calculated by subtracting the mean N2pc amplitude in the valid trials from that in the invalid trials. The validity effect for P300b was computed by subtracting the mean P300b amplitude in the invalid trials from that in the valid trials. The validity effect for the late LPC was calculated by subtracting the mean late LPC amplitude in the invalid trials from that in the valid trials, with larger values indicating greater action-related facilitation of response execution. A larger N2pc- or P300b-based validity effect in the action condition relative to the no-action condition would indicate that the arbitrary action promoted attentional selection processes. Conversely, a larger late LPC-based validity effect in the action condition relative to the no-action condition would suggest that the arbitrary action enhanced response selection processes.

#### 2.6.3 Multivariate pattern analysis

**Figure 2:**
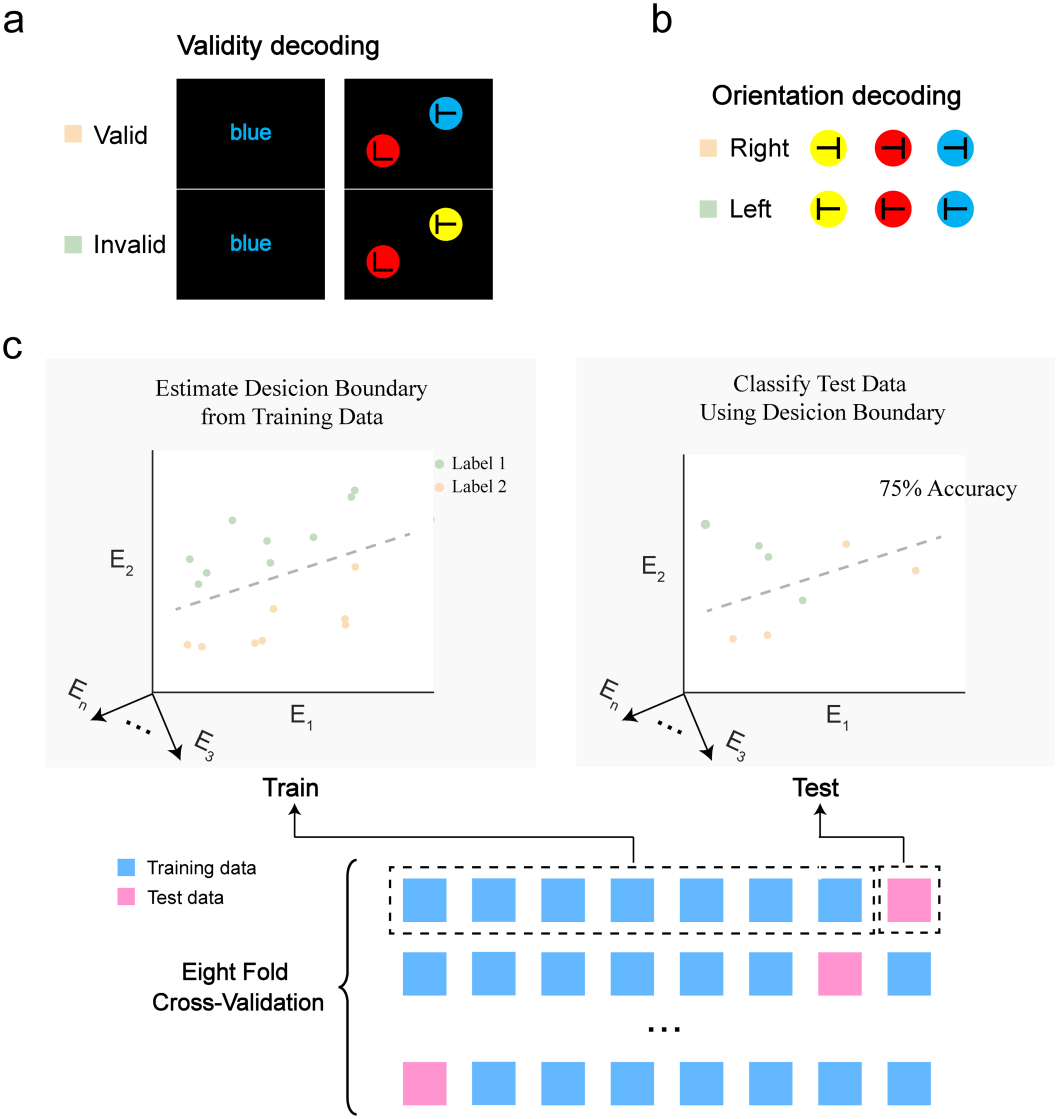
Illustration of the procedure for multivariate pattern analysis. **(a)** Decoding the validity of the search target: the “valid” label denotes that the target’s color matched the prime’s color, while the “invalid” label indicates a mismatch between the colors of the target and prime. **(b)** Decoding the orientation of the targets: the “right” label signifies that the target “T” was tilted to the right, whereas the “left” label denotes a tilt to the left. **(c)** The cross-validation procedure. E_n_ represents the nth electrode.

The multivariate pattern analysis (MVPA) consisted of two parts. First, to identify neural representations of target validity in the search task, we decoded target validity—specifically, whether the target’s color matched that of the previous prime. Successful decoding would indicate that validity information is distinguishable from neural activity patterns. This decoding was performed separately for each combination of action type and congruency. Second, to determine how the neural representation of the target’s feature is influenced by validity, we decoded the orientation of the targets, with higher decoding accuracy suggesting a more robust neural representation of the target’s feature. This decoding was conducted separately for each combination of target validity, action type, and congruency. For both decoding analyses, we employed a similar approach. Specifically, each EEG epoch was down-sampled to 100 Hz by averaging adjacent time points, enhancing the stability of the decoding process. For validity decoding, we used ‘valid’ and ‘invalid’ as labels, while for orientation decoding, the labels “left” and “right” were assigned. To improve the signal-to-noise ratio, segments with the same label were randomly grouped into eight bins and averaged within each bin. A linear support vector machine (SVM) classifier was trained for each participant at each time point, using data from all 60 electrodes, via MATLAB’s fitcsvm() function. Separate datasets were utilized for training and testing at each time point to ensure unbiased evaluation. To reduce the influence of the trial assignments and stabilize the results, we implemented eight-fold cross-validation (see Figure 2 for an illustration) and repeated the process 100 times per time point. Decoding accuracy was assessed by comparing predicted labels with actual labels. To assess the statistical significance of the decoding results, we performed a non-parametric cluster-based permutation test.

## 3 Results

### 3.1 RTs

**Figure 3:**
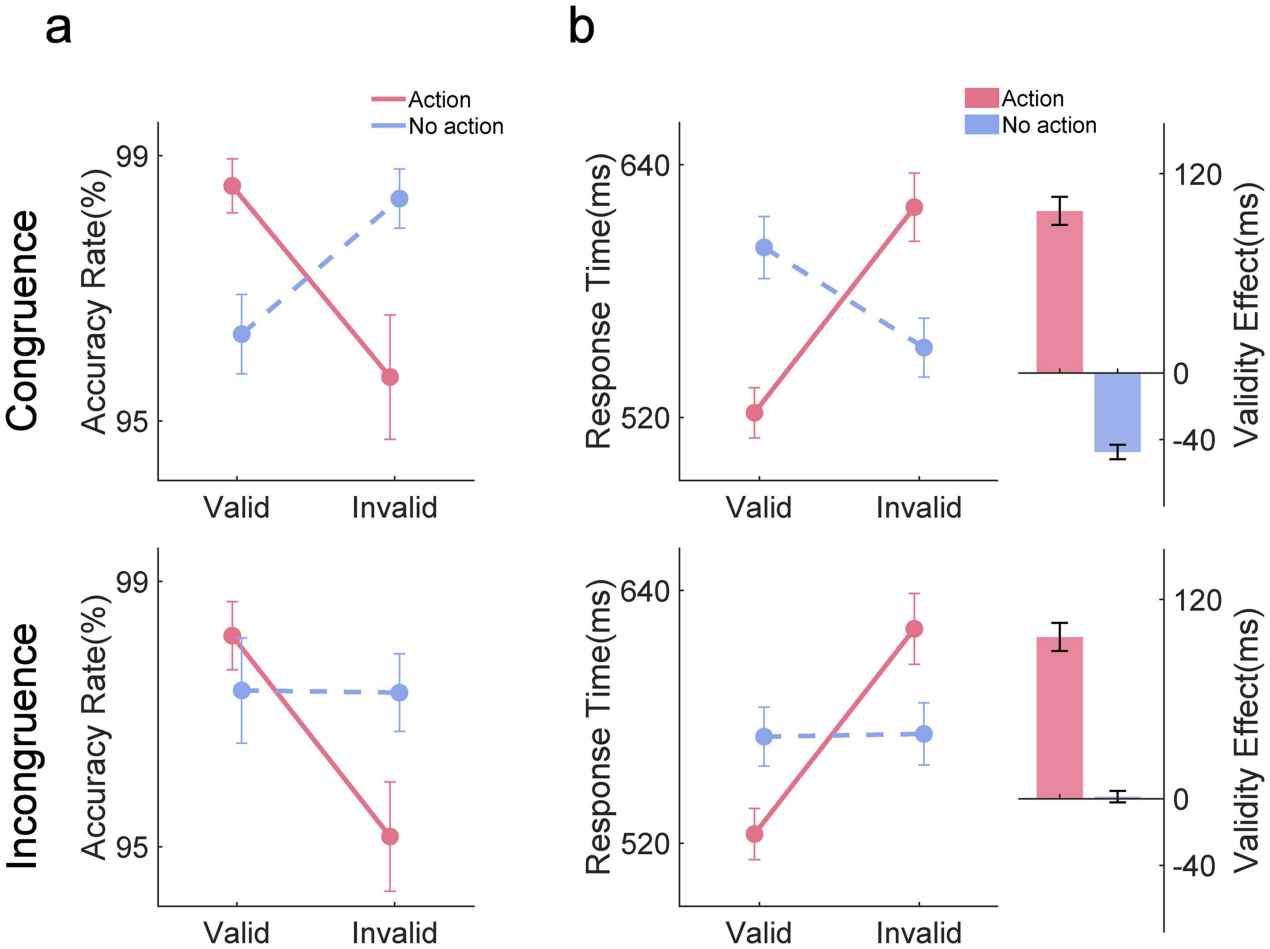
Behavior results. **(a)** Mean accuracy rate across all conditions. **(b)** Mean response times across all conditions. The validity effect was derived by subtracting the mean RTs in the valid condition from those in the invalid condition. The error bar represents the standard error.

The mean RTs across all conditions are shown in Figure 3b. With RTs as the dependent measure, we observed a main effect of validity, *F* (1,25) = 101.478, *p* < 0.001, *η^2^_p_* = 0.201, with faster responses in the valid condition than in the invalid condition (valid: 554 ± 65 ms vs. invalid: 592 ± 72 ms). The two-way interaction effects of congruence by action type, *F* (1,25) = 5.926, *p* < 0.05, *η^2^_p_* = 0.002; congruence by validity, *F* (1,25) = 37.408, *p* < 0.001, *η^2^_p_* = 0.022; and action type by validity, *F* (1,25) = 171.253, *p* < 0.001, *η^2^_p_* = 0.529, were all significant. Crucially, there was a significant three-way interaction effect, *F* (1,25) = 34.068, *p* < 0.001, *η^2^_p_* = 0.022. To follow up on this interaction, simple interaction effects were tested. Specifically, we performed separate two-way repeated-measures ANOVAs with action type (action vs. no action) and validity (valid vs. invalid) as factors for the congruence and incongruence conditions, respectively. In the congruence condition, we found a main effect of validity, *F* (1,25) = 44.403, *p* < 0.001, *η^2^_p_* = 0.085, with faster responses in the valid condition than in the invalid condition (valid: 561 ± 67 ms vs. invalid: 586 ± 75 ms). The interaction effect between action type and validity was significant, *F* (1,25) = 169.892, *p* < 0.001, *η^2^_p_* = 0.713, indicating the occurrence of an action effect. The validity effect was confirmed in the action condition (valid: 522 ± 59 ms vs. invalid: 620 ± 81 ms, *t* (1,25) = 14.532, *p* < 0.001, *Cohen’s d* = 1.338), while a reversed validity effect was observed in the no-action condition (valid: 601 ± 74 ms vs. invalid: 553 ± 70 ms, *t* (1,25) = 7.082, *p* < 0.001, *Cohen’s d* = 0.652). In the incongruence condition, there was a significant main effect of validity, *F* (1,25) = 115.573, *p* < 0.001, *η^2^_p_* = 0.395, as well as a significant interaction effect between action type and validity, *F* (1,25) = 113.806, *p* < 0.001, *η^2^_p_* = 0.374. Simple effects analysis revealed that the responses were faster in the valid condition than in the invalid condition only when an action was previously conducted (valid: 524 ± 61 ms vs. invalid: 622 ± 84 ms, *t* (1,25) = 15.145, *p* < 0.001, *Cohen’s d* = 1.316). The responses were equivalent between the valid and invalid trials in the no-action condition (valid: 571 ± 70 ms vs. invalid: 572 ± 74 ms, *t* (1,25) = 0.2, *p* = 1, *Cohen’s d* = 0.017).

We further compared the validity effect between the congruence and incongruence conditions. The results showed that following an action, the validity effect was comparable between the two conditions (congruence: 98 ± 42 ms vs. incongruence: 97 ± 42 ms, *t* (1,25) = 0.025, *p* = 0.98, *Cohen’s d* = 0.005). However, a significant difference was found when no action was executed (congruence: −48 ± 22 ms vs. incongruence: 1 ± 47 ms, *t* (1,25) = −9.583, *p* < 0.001, *Cohen’s d* = −1.879), which was due to the reversed validity effect in the congruence condition. The results suggested that the action’s prioritization of the features of the prime was not influenced by cognitive load.

With accuracy rate as the dependent measure (see Figure 3a), we found that the main effect of validity was significant, *F* (1,25) = 8.218, *p* < 0.01, *η^2^_p_* = 0.032, with a higher accuracy rate in the valid condition than in the invalid condition (valid: 0.976 vs. invalid: 0.966). The interaction between action type and validity was significant, *F* (1,25) = 18.987, *p* < 0.001, *η^2^_p_* = 0.131. Simple effects analysis revealed that the accuracy rate was higher in the valid trials than in the invalid trials in the action condition (valid: 0.984 vs. invalid: 0.954, *t* (1,25) = 5.206, *p* < 0.001, *Cohen’s d* = 0.873). While in the no-action condition, the accuracy rate was numerically lower in the valid trials than in the invalid trials (valid: 0.968 vs. invalid: 0.978, *t* (1,25) = 1.771, *p* = 0.499, *Cohen’s d* = 0.297). The results of accuracy were consistent with the insights obtained from RT analysis.

### 3.2 N2pc

**Figure 4:**
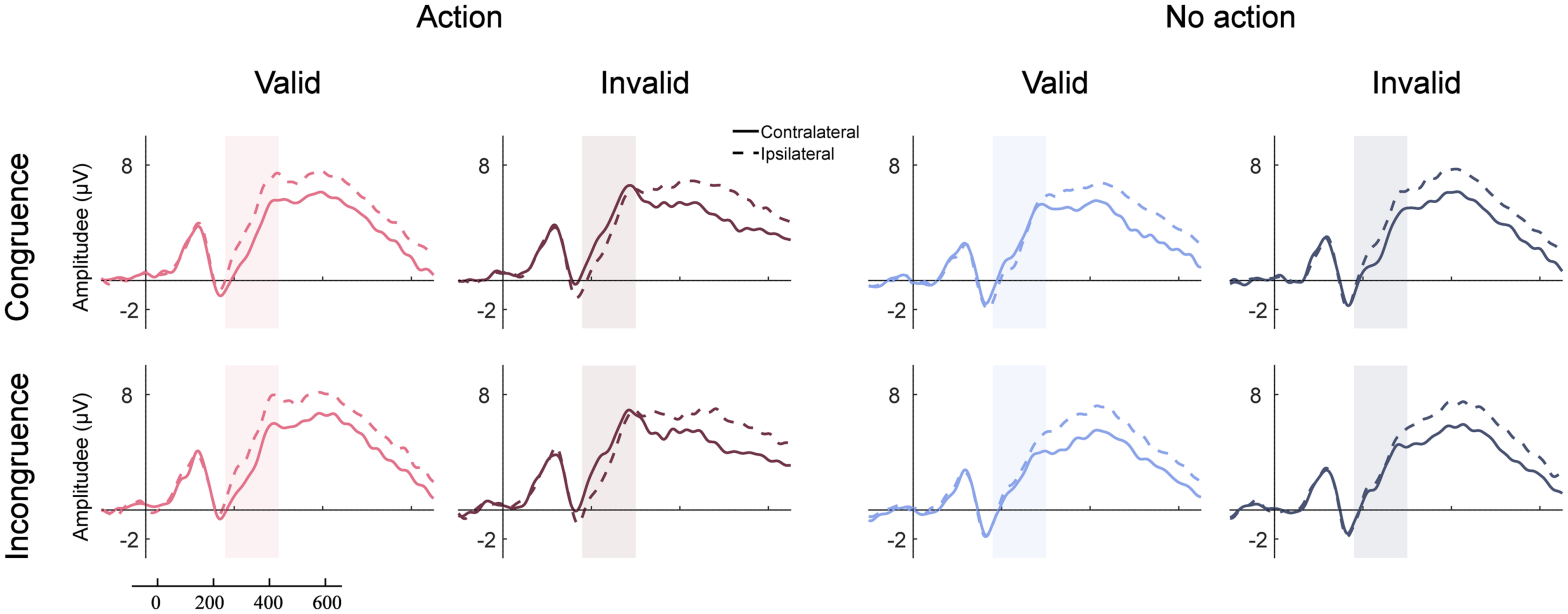
Original N2pc waves across all conditions. The shallowed areas represent the time windows of the N2pc component (180 ms to 300 ms relative to search array onset).

**Figure 5:**
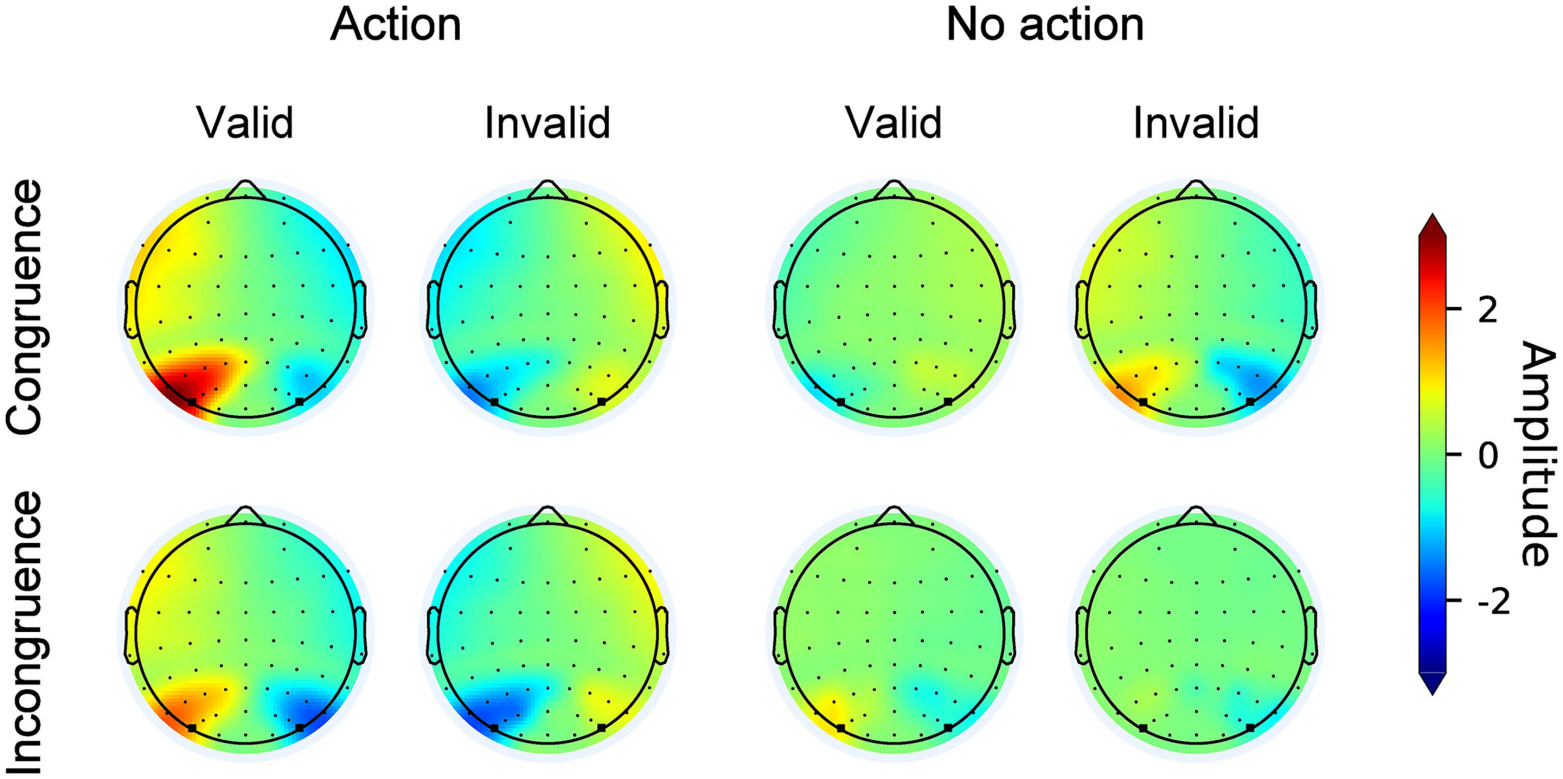
Topographic maps of the N2pc component across all conditions. Circular dots indicate recording electrodes. Electrodes used for the N2pc analysis (PO7, PO8) are marked with squares.

**Figure 6:**
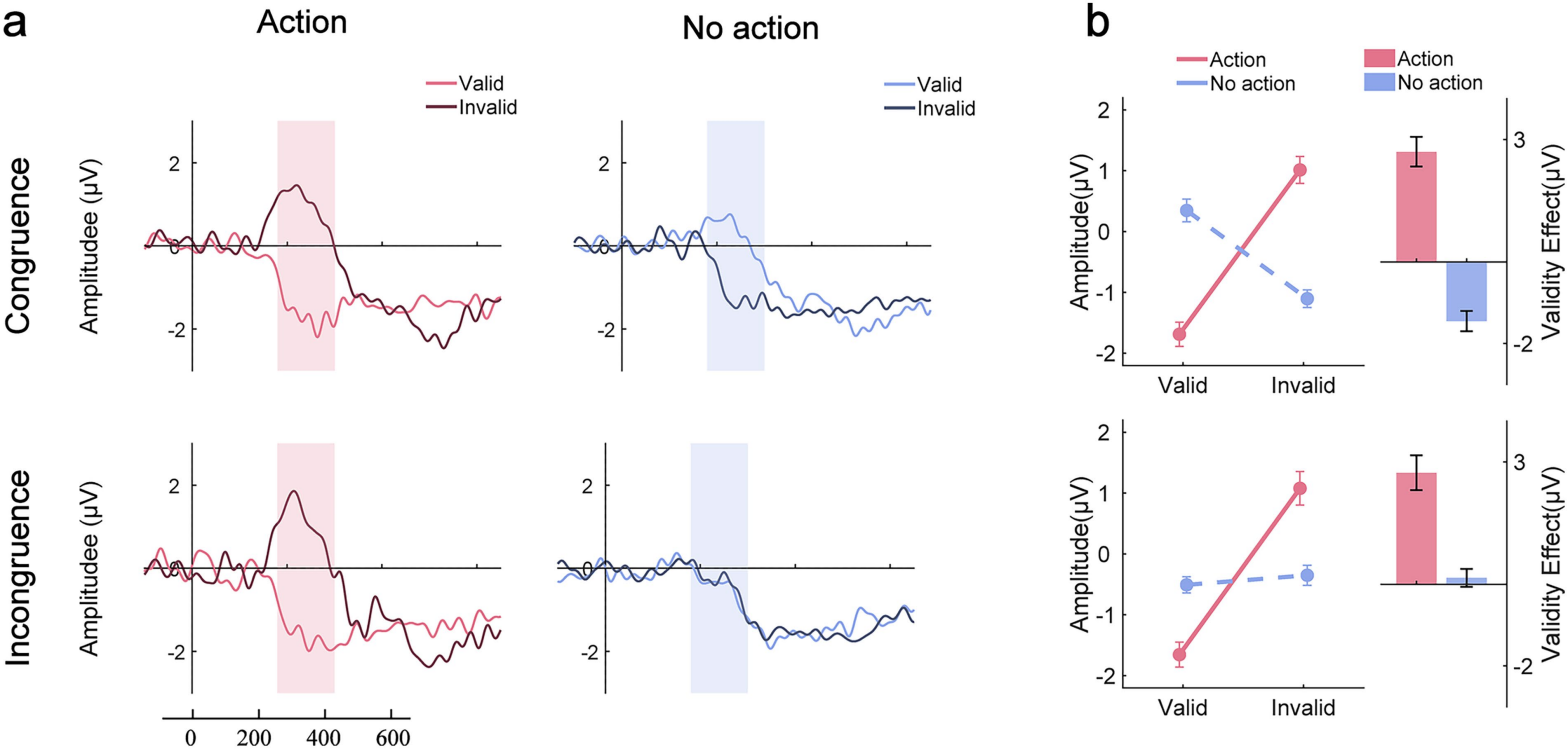
N2pc difference waves. **(a)** N2pc difference waves across all conditions. The shallowed area represents the time window of the N2pc component (180 to 300 ms). **(b)** Mean N2pc amplitude across all conditions. The validity effect was derived by subtracting the mean N2pc amplitude in the valid condition from that in the invalid condition. The error bar represents the standard error.

Figure 4 illustrates the original N2pc waves elicited by search displays from electrodes PO7/PO8 contralateral and ipsilateral to the target across all conditions. The topographic maps of the N2pc difference waves are shown in Figure 5. The results of the N2pc difference waves are shown in Figure 6a.

Using the mean N2pc amplitude as the dependent measure (Figure 6b), three-way repeated-measures ANOVA with within-subject factors of congruency (congruence or incongruence), action type (action or no action), and validity (valid or invalid) revealed that the main effect of validity was significant, *F* (1,25) = 44.471, *p* < 0.001, *η^2^_p_* = 0.14. The two-way interaction effects of congruency by validity, *F* (1,25) = 17.963, *p* < 0.001, *η^2^_p_* = 0.022, and action type by validity, *F* (1,25) = 45.705, *p* < 0.001, *η^2^_p_* = 0.369, were both significant. Crucially, the three-way interaction effect was also significant, *F* (1,25) = 15.292, *p* < 0.001, *η^2^_p_* = 0.02. Simple interaction effects analyses were conducted to reveal the nature of the significant three-way interaction. We performed separate two-way repeated-measures ANOVAs with action type (action vs. no action) and validity (valid vs. invalid) as factors for the congruent and incongruent conditions, respectively. In the congruence condition, the main effect of validity was significant, with a larger N2pc amplitude induced in the valid trials than in the invalid trials, *F* (1,25) = 19.473, *p* < 0.001, *η^2^_p_* = 0.051. The interaction effect of action type by validity was also significant, *F* (1,25) = 56.2, *p* < 0.001, *η^2^_p_* = 0.558. Following an action, the N2pc amplitude was larger in the valid condition than in the invalid condition (valid: −1.69 ± 1 μV vs. invalid: 1.01 ± 1.11 μV, *t* (1,25) = −8.684, *p* < 0.001, *Cohen’s d* = −2.777). When no action was previously executed, the N2pc amplitude was smaller in the valid condition than in the invalid condition (valid: 0.34 ± 0.93 μV vs. invalid: −1.1 ± 0.73 μV, *t* (1,25) = 4.661, *p* < 0.001, *Cohen’s d* = 1.49). In the incongruence condition, a significant main effect of validity, *F* (1,25) = 44.613, *p* < 0.001, *η^2^_p_* = 0.291, and a significant interaction between action type and validity, *F* (1,25) = 24.632, *p* < 0.001, *η^2^_p_* = 0.232, were observed. Following an action, the amplitude of the N2pc component was larger in the valid condition than in the invalid condition (valid: −1.65 ± 1.03 μV vs. invalid: 1.08 ± 1.38 μV, *t* (1,25) = −8.089, *p* < 0.001, *Cohen’s d* = 2.651). The amplitude of N2pc was comparable between the valid and invalid trials in the no-action condition (valid: −0.51 ± 0.67 μV vs. invalid: −0.35 ± 0.83 μV, *t* (1,25) = −0.465, *p* = 0.644, *Cohen’s d* = 0.152). We further found that following an action, the validity effect between the congruence and incongruence conditions was equivalent (congruence: 2.7 ± 1.8 μV vs. incongruence: 2.73 ± 2.12 μV, *t* (1,25) = −0.111, *p* < 0.0.913, *Cohen’s d* = −0.022). However, a difference was observed in the action condition (congruence: −1.45 ± 1.26 μV vs. incongruence: 0.16 ± 1.1 μV, *t* (1,25) = −6.164, *p* < 0.0.913, *Cohen’s d* = −1.209).

Overall, the N2pc results aligned with the behavioral finding. The faster RTs in the valid condition relative to the invalid condition resulted from the enhanced selection priority of the features of the previously acted-on prime. More crucially, cognitive load did not disrupt the action-induced enhancement of attention selection.

### 3.3 P300b

**Figure 7:**
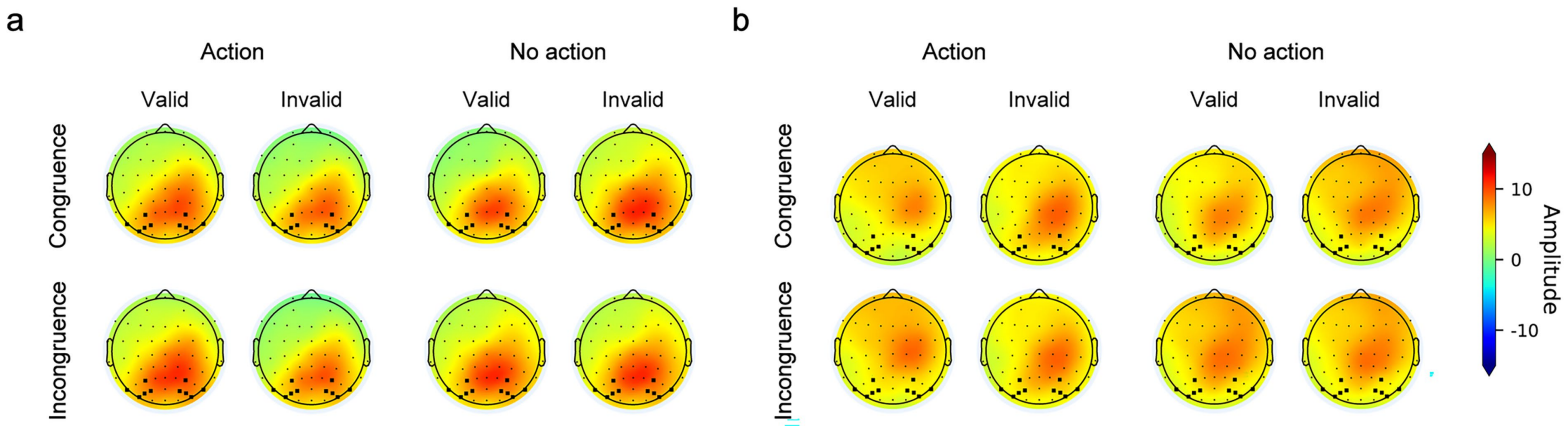
**(a)** Topographic maps of the P300b component across all conditions. **(b)** Topographic maps of the late LPC component across all conditions. Circular dots indicate recording electrodes. Electrodes used for LPC analysis (P3, P4, P7, P8, PO3, PO4, PO5, PO6, PO7, and PO8) are marked with squares.

**Figure 8:**
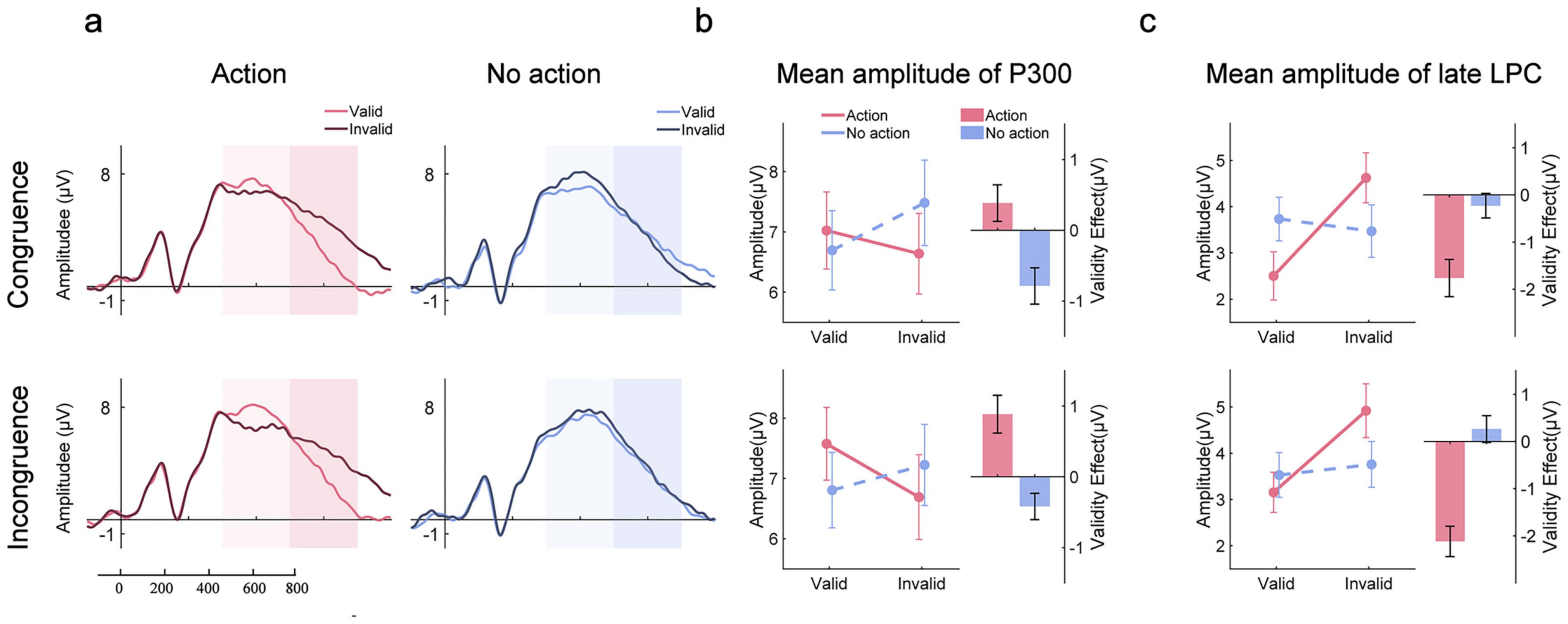
LPC waves. **(a)** The LPC waves across all conditions. The shallowed area represents the time window of the P300 component (300 to 500 ms) and the late LPC component (500 to 700 ms). **(b)** Mean amplitude of the P300 component. The validity effect was derived by subtracting the mean P300b amplitude in the invalid condition from that in the valid condition. **(c)** Mean amplitude of the late LPC. The validity effect was derived by subtracting the mean late LPC amplitude in the invalid condition from that in the valid condition. The error bar represents the standard error.

The topographic maps of the P300b component are shown in Figure 7a. In Figures 8a,b, the waveforms and the mean amplitude of P300b are illustrated. The three-way repeated-measures ANOVA with within-subject factors—congruency (congruence or incongruence), action type (action or no action), and validity (valid or invalid)—revealed that the interaction of congruency by validity, *F* (1,25) = 8.585, *p* < 0.01, *η^2^_p_* = 0.01, was significant. The validity effect was larger in the incongruence condition than in the congruence condition. Crucially, the interaction between action type and prime validity was significant, *F* (1,25) = 29.019, *p* < 0.001, *η^2^_p_* = 0.077. Following an action, the amplitude of P300b showed a larger validity effect in the action trials than in the no-action trials. The three-way interaction and all other effects were non-significant, all Fs (1,25) < 3.815, all ps > 0.062. Further analysis showed that following an action, the validity effect between the congruence and incongruence conditions had no significant difference (congruence: 0.38 ± 1.29 μV vs. incongruence: 0.88 ± 2.12 μV, *t* (1,25) = 3.423, *p* = 0.573, *Cohen’s d* = −0.402). The same pattern was observed in the action condition (congruence: −0.79 ± 1.28 μV vs. incongruence: −0.43 ± 0.93 μV, *t* (1,25) = −1.254, *p* = 1, *Cohen’s d* = −0.296).

The P300b results suggested that more attentional resources were allocated to the target when its color was the same as that of the prime following an action. This pattern was not changed by cognitive load.

### 3.4 Late LPC

The topographic maps of the late LPC component are shown in Figure 7b. In Figures 8a,c, the waveforms and the mean amplitude of the late LPC component are illustrated. Using the mean amplitude of the late LPC component as the dependent measure, the three-way repeated-measures ANOVA with within-subject factors of congruency (congruence or incongruence), action type (action or no action) and validity (valid or invalid) revealed that the main effect of validity was significant, *F* (1,25) = 22.039, *p* < 0.001, *η^2^_p_* = 0.112. The amplitude of the LPC component was larger in the invalid trials than in the valid trials (valid: 3.23 ± 2.08 μV vs. invalid: −4.19 ± 2.52 μV). The interaction between action type and validity was significant, *F* (1,25) = 38.995, *p* < 0.001, *η^2^_p_* = 0.116. Following an action, the amplitude of the LPC component was smaller in the valid trials compared to the invalid trials (valid: 2.83 ± 2.32 μV vs. invalid: 4.77 ± 2.69 μV, *t* (1,25) = 7.523, *p* < 0.001, *Cohen’s d* = 0.74). No such difference was found in the no-action condition (valid: 3.63 ± 2.3 μV vs. invalid: 3.61 ± 2.6 μV, *t* (1,25) = 0.067, *p* = 1, *Cohen’s d* = 0.007). All other main effects and interaction effects were not significant, all *Fs* (1,25) < 2.6, all *ps* >0.119.

The results of the late LPC component suggested that following an action, the efficacy of response selection was decreased in the valid trials compared to the invalid trials, which was inconsistent with the RT pattern. Nevertheless, the magnitude of the effect was still immune to the manipulation of cognitive load.

### 3.5 Multivariate pattern classification

#### 3.5.1 Decode the validity of the target

**Figure 9:**
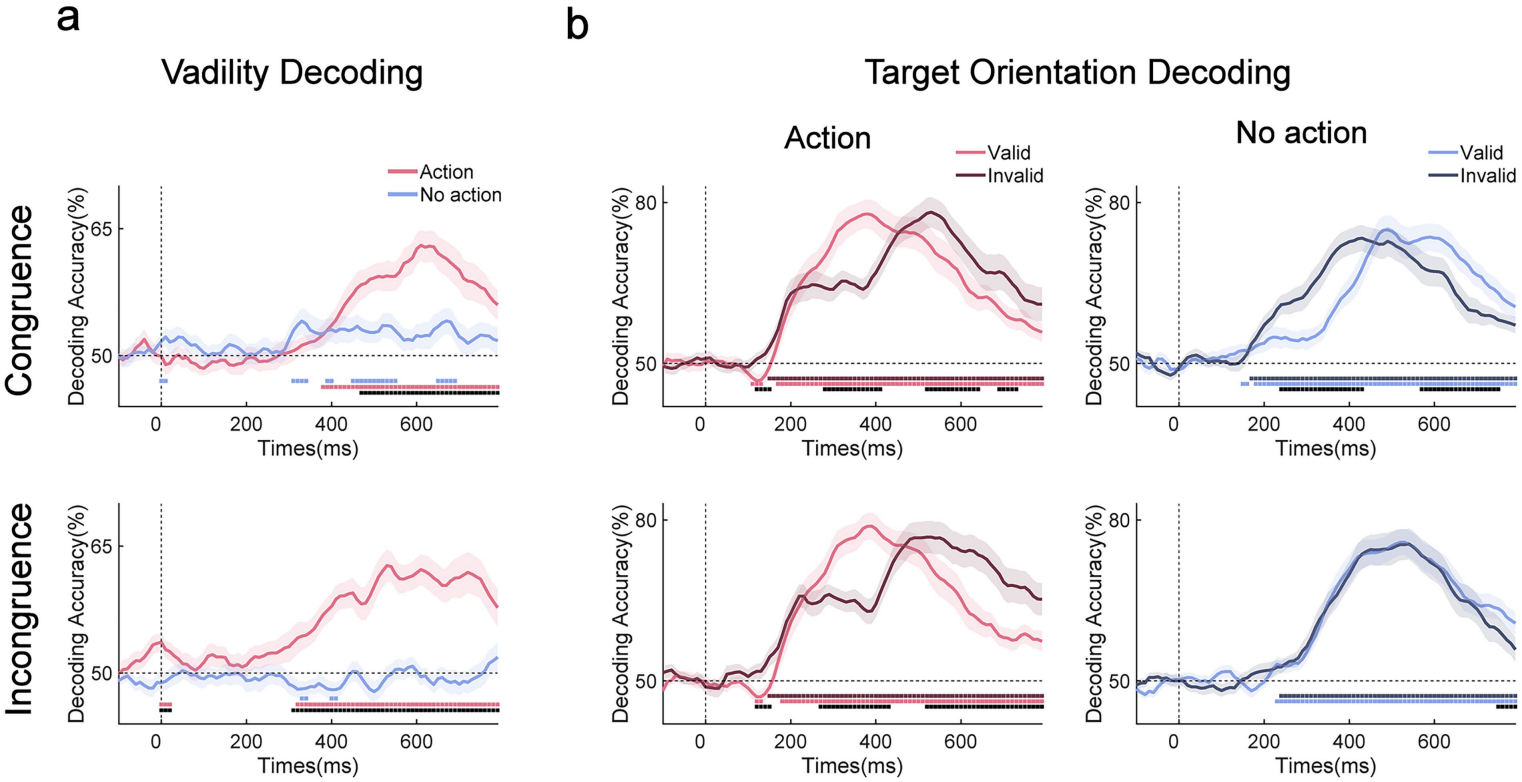
Decoding results. **(a)** Accuracy of decoding target validity. Successful decoding reflects the neural representation of the relationship (same or different) between the color of the target and the prime. **(b)** Accuracy of decoding target orientation. Successful decoding indicates the neural representation of the target’s features (orientation). Higher decoding accuracy corresponds to a stronger neural representation. The colored horizontal bars represent the time period during which decoding accuracy significantly differed from the chance level. The black horizontal bars represent the time period during which the difference in decoding accuracy was significant between the conditions.

We decoded target validity (whether the target matched the color of the previously presented prime) from neural signals during the search array presentation to identify neural markers of validity representation (see Figure 9a). Decoding accuracy was compared between action and no action conditions to examine how prior action modulated these neural representations. We found that in the congruence condition, following an action, the validity of the trials was successfully decoded, 380–790 ms, *summed t* = 207.242, *clustered p* < 0.001. The decoding was also successful in the no-action condition, 450–550 ms, *summed t* = 25.437, *clustered p* < 0.001; 650–690 ms, *summed t* = 12.123, clustered *p* < 0.001. Crucially, the decoding performance was better in the action trials than in the no-action trials, 470–790 ms, *summed t* = 110.92, *clustered p* < 0.001. In the incongruence condition, validity was successfully decoded only in the action trials, 320–790 ms, *summed t* = 249.739, *clustered p* < 0.001. In addition, the difference in decoding accuracy between the congruence and incongruence conditions reached significance during 310–790 ms, *summed t* = 227.694, *clustered p* < 0.001.

#### 3.5.2 Decode the orientation of the target

To reveal how action influences the neural representation of stimuli sharing the same or different features with the acted-on stimuli, we decoded the feature (orientation) of the target. The orientation of the target was successfully decoded approximately 200 ms after search array onset (see Figure 9b). In the congruence condition, following an action, decoding accuracy was higher in the action trials than in the no-action trials during the early stage of the search, 280–410 ms, *summed t* = 53.318, *clustered p* < 0.001, while it was lower during the late stage, 520–640 ms, *summed t* = 36.405, *clustered p* < 0.001; 690–730 ms, *summed t* = 11.775, *clustered p* < 0.001. When no action was previously executed, decoding accuracy was higher in the no-action trials than in the action trials during the early stage, 240–430 ms, *summed t* = 70.265, *clustered p* < 0.001, but it was lower during the late stage, 570–750 ms, *summed t* = 66.325, *clustered p* < 0.001. The results are reasonable, considering the opposite patterns observed between the N2pc and late LPC components. In the incongruence condition, following an action, decoding accuracy was higher in the action trials than in the no-action trials during the early stage, 270–430 ms, *summed t* = 68.602, *clustered p* < 0.001, but it was lower during the late stage, 520–790 ms, *summed t* = 107.014, *clustered p* < 0.001. When no action was previously executed, decoding accuracy was basically equal; however, a slight discrepancy was detected during the very late stage, 750–790 s, *summed t* = 14.494, *clustered p* < 0.001.

## 4 Discussion

The goal of the present study was to investigate whether the action effect is influenced by cognitive load. In our experiment, we changed the congruency between the color and the word meaning of the prime to manipulate cognitive load. The results showed that the prime was prioritized by action, as evidenced by the shorter response times when the target’s color matched the prime’s color compared to when they mismatched, consistent with previous studies (Han et al., 2020; Robinson et al., 2018; Suh and Abrams, 2018; Wang et al., 2017; Wang et al., 2021; Weidler and Abrams, 2014, 2016, 2018; Weidler et al., 2018). Our results indicated that the attentional priority of the prime’s features was enhanced following an action, as evidenced by the larger N2pc amplitude in the valid trials than in the invalid trials when an action was executed. Surprisingly, we found that response efficacy decreased in the valid trials compared to the invalid trials. The decoding results converged with the patterns of the RT and ERP results. On the one hand, the successful decoding of the validity of the target in the action condition further provided a neural marker of the action effect. Moreover, for orientation decoding, we found that, following an action, the neural representation of the target was better in the early stage (corresponding to the time window of attention) but was worse in the late stage (corresponding to the time window of response selection). Importantly, the results of the RT, ERP, and decoding were all similar between the congruency and incongruency conditions, which suggested that the effect of action in perception was not influenced by cognitive load.

The crucial finding of the current study was that the prioritization of the feature of the acted-on stimulus was not reduced by the cognitive load induced by the incongruence between the color and the meaning of the prime. As stated before, an automatic process should be insensitive to task relevance and should not consume cognitive resources. Previous studies demonstrated that the action effect still occurred when the feature of the prime was not relevant to both the search task and the action task. In these studies, instructions directly informed participants whether they should act or not (e.g., “go” or “no-go”), so they did not need to process the features of the prime (Weidler and Abrams, 2014, 2016, 2018). The survived action effect in the situation suggests that it is insensitive to task relevance. Combined with our finding that the validity effect induced by action was unaffected by cognitive load, the current evidence suggests that the action’s prioritization of the prime may involve an automatic process.

Our results provide insights into the cognitive mechanism underlying the action effect. For the first time, we provide neural evidence supporting that action enhances the attention priority of acted-on stimuli. Enhanced attention to the target that shared the color of the previous prime led to better search performance in the valid trials than in the invalid trials. This finding is in line with a previous eye-tracking study (Weidler et al., 2018), which also reported that, following an action, search efficacy was improved in valid trials. Nevertheless, action prolonged response time when targets shared the same color as the prime, which contradicts the faster RTs observed in the valid trials following an action. Although surprisingly, the finding should not be occasional. A similar pattern was also observed by Weidler et al. (2018). They found that the late stage of the search—the time from fixation on the target to the response—was longer in valid trials than in invalid trials when an action was previously executed.Although action facilitated search and interfered with the response process, the former played a more dominant role, leading to an overall behavioral benefit in valid trials.

Some researchers have proposed that the action effect could be explained by event-file theory (Wang et al., 2017; Weidler and Abrams, 2014). The assumption is that actions are integrated together with the representations of acted-on objects in working memory. Wang et al. (2017) conducted experiments to examine this possibility. In their experiment, participants did not respond by pressing a key after finding the target, so the integration of the previous action and key press should not have influenced the performance of the search task. However, they still observed the action effect, suggesting that it cannot be fully explained by event-file theory. In addition, one would expect response speed to be quicker in valid trials relative to invalid trials following an action if the integration of the color of the prime with the key press facilitated later response processes. This was not supported by our results, as the amplitude of the LPC component, indexing the efficacy of response selection, was smaller in the valid trials than in the invalid trials following an action.

A previous study argued that the action effect might be a special type of selection history effect (Weidler and Abrams, 2016). Selection history is a third source of attentional control, alongside goal-driven and stimulus-driven attention, emphasizing the role of past experience in attention selection (Anderson et al., 2011a, 2011b, 2021; Awh et al., 2012). In the context of the action effect, the experience refers to people’s selection of a certain feature of the prime (people only pressing the key when encountering a specific feature). The selection of the feature might promote the attention selection of similar features in the immediate search task, similar to the effect of priming. Moreover, selection history has also been found to be insensitive to cognitive load (Gao and Theeuwes, 2020; Vicente-Conesa et al., 2022), which is in line with what we observed in the action effect. If this is the case, the action effect might reveal a novel form of previous experience in feature selection that influences the attention selection of similar features by simply performing an arbitrary action.

Although the current study deepened our understanding of the action effect, there were several limitations in our experiment. First, the search task in our experiment was relatively simple, with only two items presented in the search array. Given this, the requirement for cognitive resources may be low, and it was unknown whether the manipulation of cognitive load using the Stroop effect was sufficient to induce a considerable limitation of available cognitive resources. Note that this limitation also existed in previous research providing evidence supporting the biased-competition explanation of the action effect (Huffman and Pratt, 2017). Further studies should adopt more demanding search tasks to explore the influence of cognitive load on the action effect. Another limitation of the current study is the timing of cognitive load induction. In our experiment, cognitive load was induced when the action was executed. This manipulation allowed us to examine the influence of cognitive load on the prioritization of the prime by the action. However, it is also possible that the expression of the action’s prioritization requires the engagement of cognitive resources. For example, the magnitude of the action effect might be influenced when cognitive load is induced during the search task. Future studies should further examine this possibility.

Investigating the mechanisms underlying action effects holds not only important theoretical significance for revealing how action and cognition interact but also substantial potential for practical applications. The finding that arbitrary actions can enhance search performance suggests promising applications if such action-induced improvements can be extended to more general cognitive activities. For instance, incorporating specific motor activities during learning tasks might optimize cognitive outcomes. The present study’s revelation that action effects remain intact under cognitive load provides valuable guidance for such potential applications by illuminating the boundary conditions of action effects. Specifically, our results suggest that we would expect action-induced cognitive enhancement to be equivalent regardless of how the action is performed—whether executed with full attention or while multitasking. This characteristic of action effects enhances their applicability. Nevertheless, to make such applications practical, further research is needed to address key questions, such as whether action effects generalize beyond visual search to other cognitive domains, such as memory and learning.

## 5 Conclusion

In summary, the current study revealed that the magnitude of the action effect was not influenced by cognitive load, indicating it might involve an automatic process not requiring cognitive resources. The electrophysiological data demonstrated that the cognitive mechanism underlying the action effect was an enhanced attentional priority for the features of acted-on stimuli.

## Data Availability

The raw data supporting the conclusions of this article will be made available by the authors, without undue reservation.
